# Draft Genome Sequence of Enterotoxigenic Escherichia coli Strain E24377A, Obtained from a Tribal Drinking Water Source in India

**DOI:** 10.1128/genomeA.00225-15

**Published:** 2015-04-02

**Authors:** Ashok J. Tamhankar, Sandeep S. Nerkar, Prashant P. Khadake, Dadasaheb B. Akolkar, Sachin R. Apurwa, Uday Deshpande, Smita U. Khedkar, Cecilia Stålsby-Lundborg

**Affiliations:** a Indian Initiative for Management of Antibiotic Resistance (IIMAR), Department of Environmental Medicine, R.D. Gardi Medical College, Ujjain, India; b Department of Public Health Sciences (Global Health/IHCAR), Karolinska Institutet, Stockholm, Sweden; c Life Technologies India Ltd., Thane, India; d Datar Genetics Ltd., Nashik, India; e Labindia (GOPD), Thane, India; f Bac-Test Laboratory, Nashik, India

## Abstract

Enterotoxigenic Escherichia coli (ETEC) is a major cause of diarrheal disease in humans and animals. Its dissemination can occur through water sources contaminated by it. Here, we report for the first time the draft genome sequence of ETEC strain E24377A, obtained from a tribal drinking water source in India.

## GENOME ANNOUNCEMENT

Infection by enterotoxigenic Escherichia coli (ETEC) results in a large volume of watery diarrhea, generally known as traveler’s diarrhea (1). Besides humans, the infection also causes diarrhea in animals (2). ETEC primarily colonizes the small intestine by way of the colonization factor antigen (CFA) pili; E. coli strain E24377A has been shown to contain CFA pili types CS1 and CS3. Two additional toxins are thought to be responsible for its virulence, a heat-stable enterotoxin and a heat-labile enterotoxin. Other virulence factors include those belonging to serotype O139:H28 and potential factors encoded on 6 plasmids. ETEC colonizes the lower gut of animals and survives when released into the natural environment, allowing widespread dissemination to new hosts. Since the spread can easily occur through drinking water, its presence in drinking water sources has great epidemiological and public health consequences, particularly in the case of tribal areas, where diarrheal disease management measures are largely inadequate. However, the whole-genome sequence of ETEC E24377A from drinking water sources from tribal areas has not been reported.

The complete genome sequences of three ETEC strains infecting humans, E24377A (3), H10407 (4), and B2C (5), as well as one ETEC strain, W25K, infecting piglets (6), have been published. Recently, draft whole-genome sequences of 10 serogroup O6 ETEC strains from historical and cruise ship outbreaks (7) were announced. Here, we report the draft genome sequence of strain E24377A, which is an O139:H28 serotype strain of ETEC that was obtained from an environmental sample intercepted from a drinking water well in a hilly tribal village in India. In this village, we reported an earlier occurrence of diarrheal cases and the contamination of tribal drinking water wells by fecal coliform and E. coli (8).

Genomic DNA was extracted from the E. coli O139:H28 strain E24377A using the GenElute bacterial genomic DNA kit (Sigma Aldrich), according to the manufacturer’s protocol. The genomic DNA was used to generate a library using the Ion Xpress fragment library kit and sequenced using a semiconductor-based sequencing platform (Ion Proton; Life Technologies). The sequencing generated approximately 3 million singleton reads, constituting 101-fold coverage of the genome. The reads were overlapped where possible and trimmed for quality using the PRINSEQ version 0.20.4 software. A *de novo* assembly of the overlapped and quality-trimmed reads was generated using the CLC Workbench. The final assembly consists of 453 contigs. The genome size of ETEC O139:H28 strain E24377A, intercepted from an aquatic environment, is estimated to be 4.9 Mb. The genome sequence was annotated using the RAST genome annotation server (9). An analysis of the genome showed that the environmental ETEC O139:H28 strain E24377A has 597 subsystems, 5,305 coding sequences, and 67 RNAs. Type II and type VI protein secretion systems were identified. Classical ETEC virulence genes, *eltA* and *eltB*, the genes encoding the two subunits of heat-labile enterotoxin, were also found.

### Nucleotide sequence accession numbers.

This whole-genome shotgun project has been deposited in DDBJ/EMBL/GenBank under the accession no. JXRF00000000. The version described in this paper is the first version, JXRF01000000.
